# Meta analysis of indocyanine green fluorescence in patients undergoing laparoscopic colorectal cancer surgery

**DOI:** 10.3389/fonc.2022.1010122

**Published:** 2022-10-26

**Authors:** Jia Deng, Wenting Hu, Yang Li, Kai Xiong, Tinghui Yue, Xiangquan Lai, Tianbao Xiao

**Affiliations:** ^1^ College of Clinical Medicine, Guizhou University of Traditional Chinese Medicine, Guiyang, China; ^2^ College of Pharmacy, Dali University, Dali, China; ^3^ Colorectal and Anal Surgery, The First Affiliated Hospital of Guizhou, University of Traditional Chinese Medicine, Guiyang, China

**Keywords:** indocyanine green, laparoscopic surgery, meta-analysis, colorectal cancer, systematic review

## Abstract

This meta-analysis intended to systematically evaluate the clinical implications of indocyanine green fluorescence (ICG) in patients undergoing laparoscopic colorectal surgery. PubMed, MEDLINE, Cochrane Library, EMBASE, China National Knowledge Infrastructure (CNKI), Wanfang Database, VIP Medical Information System and China Biomedical Database were synthetically searched for studies published from inception to April 14, 2022. The randomized controlled trials comparing ICG-use with controls were selected. The incidence of anastomotic leakage (AL), lymph node detection, operation duration, intraoperative bleeding, postoperative morbidity, and hospitalization time were evaluated in summary analysis, and calculated the corresponding 95% confidence intervals (CI). Subsequently, in addition to subgroup analyses, studies for heterogeneity, sensitivity, and publication bias were carried out. Consequently, 3453 patients in the enrolled 15 studies were included; 1616 patients were allocated to the experimental group, and 1837 patients were assigned to the control group. The ICG group had a significantly decreased risk of AL (RR: 0.50, 95% CI: 0.37–0.67) and shorter hospitalization time (SMD: -0.31, 95% CI: -0.54–0.08) compared to the control group. Meanwhile, the ICG showed clearly better lymph node detection (SMD: 0.19, 95% CI: 0.02–0.36). However, when the content of operation duration (SMD: -0.07, 95% CI: -0.30–0.15) and intraoperative bleeding (SMD: -0.16, 95% CI: -0.35–0.04) were compared, no statistical significance was found. Furthermore, the pooled analysis of postoperative morbidity was not statistically significant (RR:0.79, 95% CI: 0.58–1.08). The results of the subgroup analysis of AL indicated that there may be regional variations in AL (RR: 0.50, 95% CI: 0.37–0.67) but not in postoperative morbidity (RR: 0.79, 95% CI: 0.58–1.08). In conclusion, the application of ICG in laparoscopic colorectal surgery can effectively reduce the AL, lymph node detection, and hospitalization time. However, more multicenter large-sample randomized controlled trials are required to further confirm its advantages. The meta-analysis was registered in PROSPERO (no. CRD42022288054).

## Introduction

Colorectal cancer (CRC) is one of the most prevalent gastrointestinal malignancies with high incidence and mortality, seriously threatening human life and health. The latest global statistics show 1,148,515 new cases of colon cancer and 732,210 new cases of rectal cancer in 2020, with a 9.4% mortality rate for CRC patient (1). In addition, mortality from CRC is projected to increase significantly by 71.5% (rectal cancer) and 60% (colon cancer) in 2035 (2). In the Chinese population, CRC is also one of the most common cancers and has shown a pattern of increasing incidence and mortality rates over time (3). Radical surgery (R0/R1 resection) remains to be a primary procedure for the management of CRC and recommended by the National Comprehensive Cancer Network (NCCN) and European Society for Medical Oncology (ESMO) guidelines. Laparoscopic colorectal surgery has become a standard and mainstay operation for the treatment of localized CRC with the continuous development of minimally invasive technology (4, 5). In comparation with open abdominal surgery, laparoscopic surgery has significant advantages, such as minimally invasiveness, little bleeding, quickly postoperative recovery and improvement in patient care quality. However, the blindness of lymphatic clearance and the inability to touch become the biggest challenges to be solved in the laparoscopy. Furthermore, the technical challenges of laparoscopic surgery for CRC also have been raised by robotic technology with advantages of articulating wrists, lack of hand tremors, and decreasing the learning curve (6). Therefore, an auxiliary technology is urgent for laparoscopic colorectal surgery to improve its precision and individuality.

Indocyanine green (ICG) is a near-infrared light contrast agent with good biocompatibility. It is excited by external light at a wavelength of 750–800 nm, and in-turn emits near-infrared light of a longer wavelength for visualization of tissues and organs (7). Since Nagata first applied ICG in colorectal surgery in 2006, considerable research value and good application prospects in the adjuvant diagnosis and therapy of CRC have been demonstrated by this technology (8–12). More and more attention has been paid to the role of ICG in laparoscopy, including the accurate intraoperative localization of the tumor and the rigorous cleaning of the lymphatic drainage area. In addition, since ICG can be used to observe the intestinal blood supply at the anastomosis, it has significant practical advantages in lowering the incidence of postoperative anastomotic leakage (AL) (13). However, this technique is still in the exploratory stage in the diagnosis and treatment of CRC, so there are no standardized application criteria and operation specifications, and few guidelines and consensus for reference. Systematic observation and analysis are lacking in the application value of ICG. In this study, we will investigate the efficacy of ICG in laparoscopic colorectal surgery and provide reliable evidence-based medical evidence for its wide application in clinical practice.

## Materials and methods

### Protocol registration

This agreement was previously registered in PROSPERO in December 2021. (Number: CRD42022288054, https://www.crd.york.ac.uk/PROSPERO/display_record.php?RecordID=288054).

### Qualification criteria

The Cochrane Handbook for Systematic Reviews of Interventions and the PRISMA Statement were consulted for this study. Meanwhile, the “PICOS” principles were used as a guide for this study’s inclusion and exclusion standards. Studies must meet the following inclusion criteria: (i) Patients with laparoscopic colorectal surgery for CRC (including colon and rectal cancer) were of different sex, age, race, and nationality. (ii) The experimental group was allocated in laparoscopic colorectal surgery by ICG, while the control group was assigned in laparoscopic colorectal cancer without ICG. (iii) The effectiveness between conventional laparoscopic colorectal surgery and laparoscopic colorectal surgery with ICG was compared. (iv) At least one of these outcome indicators must be reported, including the main outcome indicators such as AL and lymph node detection. The secondary outcome indicators include postoperative morbidity, operation duration, hospitalization time, and intraoperative bleeding. (v) The design of these studies were prospective randomized controlled trials (RCTs). Studies were asked to meet the following exclusion criteria: (i) Full text or specific values of the required indicators were not available. (ii) Comparison of the efficacy of two procedures was not included; (iii) duplicate publications; (iv) case reports, conference reports, reviews, animal experimental papers, and meta-analysis.

### Search methodology

Two researchers conducted a search in the following databases: pubMed, MEDLINE, Cochrane Library, EMBASE, China National Knowledge Infrastructure (CNKI), Wanfang Database, VIP Medical Information System, and China Biomedical Database (CBM). Meanwhile, the period was limited from the database’s creation until April 14, 2022. The search terms were composed of the following medical themes (MeSH) and additional conditions: (colorectal cancer/colorectal neoplasms/colorectal tumor) AND (indocyanine green/fluorescence/spectroscopy near-infrared) AND (randomized controlled experiments/clinical trials). Furthermore, manual studies would be conducted to find potential references. Language was not an obstacle to publication.

### Research selection

The program Endnote™, Version X8 (Thompson Reuters) was used to combine all search results. Repeated studies were manually removed. Two researchers independently screened the original studies and then read the full text to select the literature that met the inclusion criteria. Disagreements should be settled through discussion or by reaching an agreement with a third party.

### Data selection and analysis

The data extraction was completed independently by two researchers who cross-checked the results using a uniform data extraction form. The following information was included in the data extraction: (i) The research ID, including the first author’s name and publication date. (ii) The research participant, including the number and age of the participants. (iii) The treatment program for the experimental and control groups. (iv) the main outcome indicators, such as AL and lymph node detection, and the secondary outcome indicators included postoperative morbidity, operation duration, hospitalization time, and intraoperative bleeding. The original author was contacted if the necessary information was not available, and the data was considered missing if there was no response. Furthermore, the disagreements between two researchers were settled through discussion or consensus, and a third researcher was requested to solve the conflict if required.

### Quality evaluation

The quality of the included studies was evaluated by two researchers independently, and conflicts among them were resolved through discussion or third-party consensus. Risk bias assessments were produced using RevMan 5.3 software based on the Cochrane Risk of Bias Assessment Tool. The evaluation included the following seven major components: random sequence generation; allocation concealment; blinding of patients and testers; blinding of outcomes assessors; incomplete results; selective reporting; and other bias (such as potential bias related to special research design, declaration of fraud, etc.). Finally, judgments should be made in this study, such as “low bias risk,” “high bias risk,” and “uncertain bias risk.”

### Data analysis

The data was statistically analyzed using Stata 14.0 (Stata Corporation). Relative risk (RR) and standardized mean difference (SMD) were used as effect statistics for dichotomous and continuous variables, and 95% confidence intervals (CI) were calculated. The *I* ^2^ test was initially used to investigate heterogeneity. If *P* ≥ 0.1 and *I* ^2^ ≤ 50%, heterogeneity between studies was small and a fixed-effect model was used; if *P* < 0.1 and *I* ^2^ > 50%, heterogeneity existed between studies and a random-effect model was used for meta-analysis. Subgroup analysis was carried out based on the various included study areas, and sensitivity analysis was carried out using the leave-one-out method for outcome indicators with high heterogeneity, as well as the contour-enhanced funnel plot.

## Result

### Literature selection results

**Figure 1:**
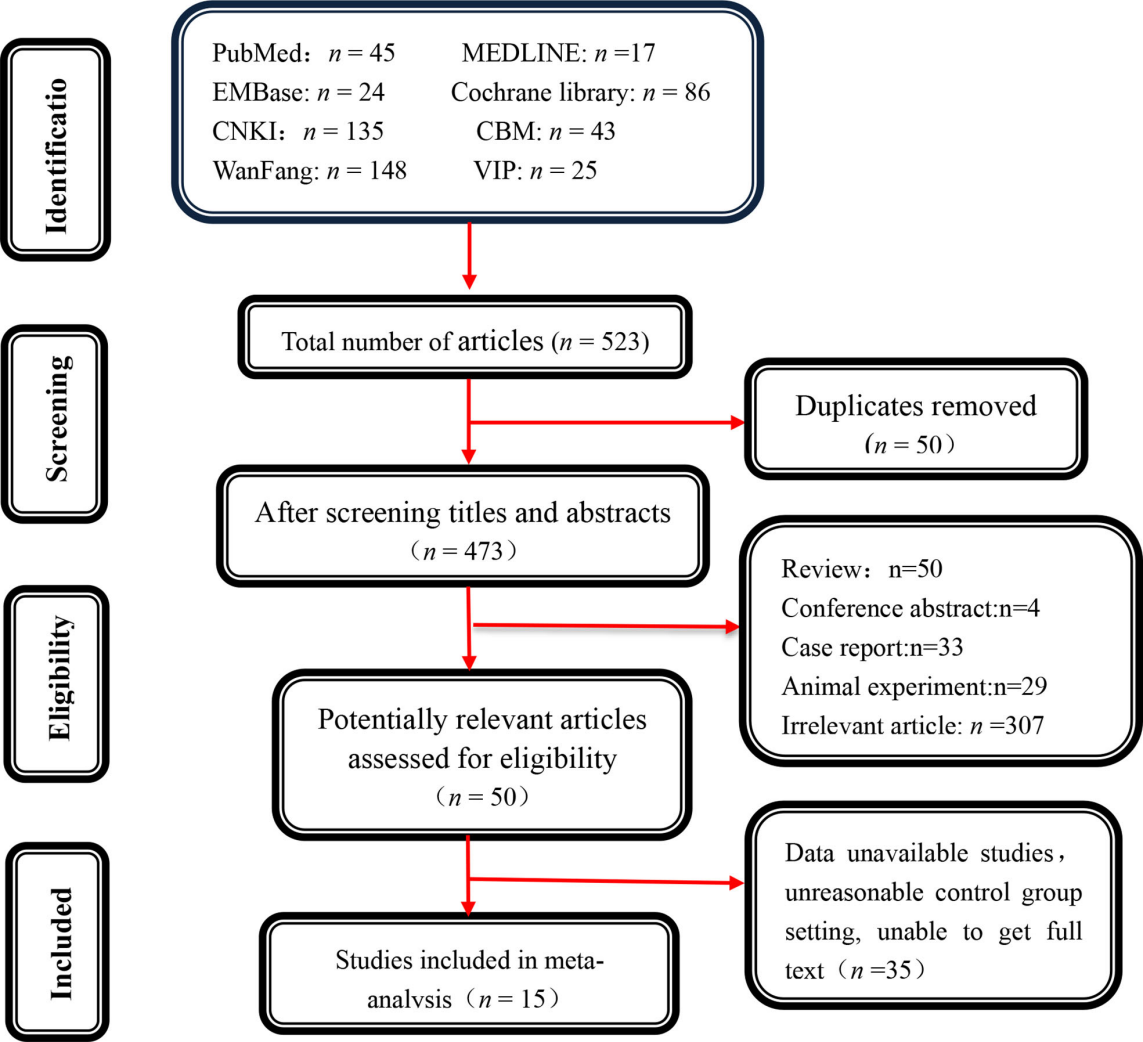
Literature screening process.

Initially, 523 potentially relevant papers were screened, and 50 duplicate items were completely removed. Furthermore, 423 papers were excluded after reading the titles and abstracts because they were not suitable for the inclusion criteria. 35 articles were retained after carefully reviewing the abstracts and full texts of the remaining 50 articles. Finally, 15 studies were enrolled after rigorous screening using inclusion and exclusion criteria (**Figure 1**).

### Study characteristics

A total of 3453 individuals (14–28) were enrolled in 15 included studies; 1616 of these patients were in the ICG group and 1837 were in the control group. The fundamental features of all included studies were displayed in **Table 1**. ICG was administered through an intravenous injection (IV) in 12 trials (14, 15, 17–21, 24–28) and as a submucosal injection in 3 trials (16, 22, 23). The AL result was reported in 10 trials (14, 15, 17–21, 25, 27, 28). There were 5 trials that reported lymph node detection (22–24, 26, 28). Postoperative morbidity was observed in 8 trials (14–16, 22–24, 26, 28). Operation time was reported by 9 trials (16, 21–28), hospitalization time was reported by 8 trials (16, 19, 21–24, 26, 28), and intraoperative bleeding was reported by 7 trials (21–24, 26–28).

**Table 1:** Basic characteristics of the included studies in the meta-analysis.

Research ID	Sample number (n)			Ages (year)		Tumor types	Dose of ICG	Route of medication	Outcomes
		study	control		study					control
Alekseev M (14)	187		190	Range:21-86	Range:66-85	CRC	0.2 mg/kg	iv	①③
De Nardi, P (15)	118		122	Range: 29-88		CRC	0.3 mg/kg	iv	①③
Park JH (16)	114		228	67.91 ± 8.94	66.81 ± 10.18	CRC	0.5-1 ml	submucosal injection	③④⑤
Ishii M (17)	223		265	Range:30–90	Range:27–93	CRC	5 mg	iv	①
Bonadio L (18)	33		33	71.85 ± 11.1	69.03 ± 11.3	CRC	0.2 mg/kg	iv	①
Kin C (19)	173		173	58.2 ± 13.2	58.1 ± 13.2	CRC	3 ml	iv	①⑤
Ren P (20)	63		82	NR		CRC	2.5 mg/ml	iv	①
Wu G.C (21)	130		130	66.63 ± 4.72	67.53 ± 4.59	CRC	2.5 mg/ml	iv	①④⑤⑥
Zhang J.F (22)	68		77	60.2 ± 12.5	58.4 ± 14.1	CRC	2.5 mg/ml	submucosal injection	②③④⑤⑥
Zhou SC (23)	12		30	60.3 ± 9.6	58.5 ± 9.5	RC	0.1 mg/ml	submucosal injection	②③④⑤⑥
Su H (24)	84		105	59.1 ± 11.1	60.2 ± 9.8	CC	3 ml	iv	②③④⑤⑥
Tsang YP (25)	62		69	69.82 ± 9.89	67.71 ± 11.65	CRC	10 mg	iv	①④
Ge L (26)	36		22	56.4 ± 9.6	58.1 ± 11.0	CC	3 ml	iv	②③④⑤⑥
Foo CC (27)	253		253	66.6 ± 10.6	67.2 ± 11.0	CRC	5 mg	iv	①④⑥
Jing D.S (28)	60		58	56.7 ± 7.42	54.4 ± 7. 64	CC	2.5 mg/ml	iv	①②③④⑤⑥

NR, not reported; IV, intravenous injection; ①, AL; ②, Lymph node detection; ③,Postoperative morbidity; ④,Operation duration;⑤, Hospitalization time; ⑥, Intraoperative bleeding.

### Quality evaluation of included studies

**Figure 2:**
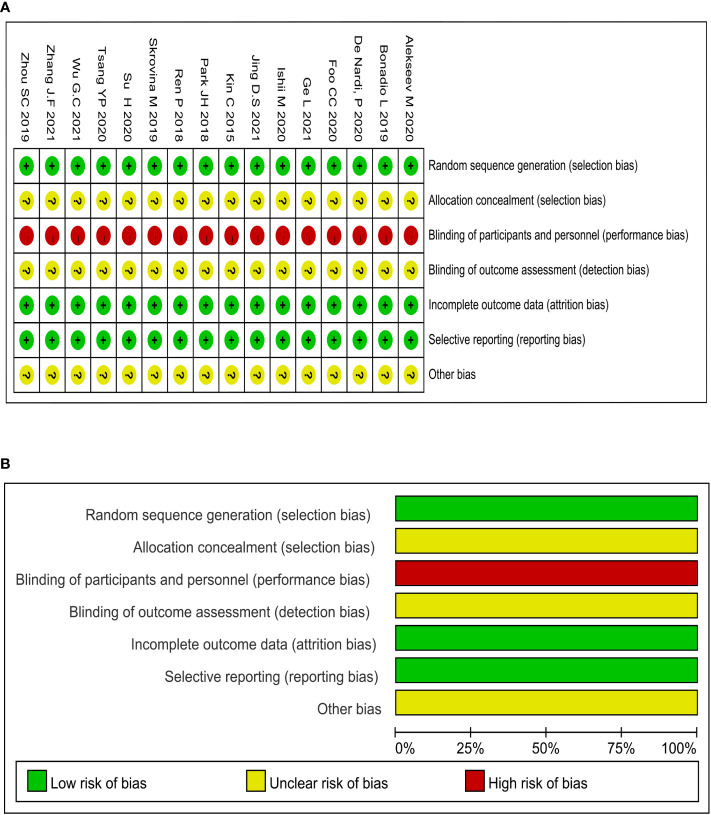
Methodological quality graphs and summaries: **(A)** Summary of risk of bias; **(B)** Graph of risk of bias.

The methodological quality assessment of the 15 included studies was displayed in **Figure 2**. The formation of random sequences was fully recognized in all of the selected studies, as shown in **Figure 2A**. Meanwhile, allocation concealment was ambiguous. None of studies mentioned the application of blinding, and the evaluation of performance bias was regarded as high risk (**Figure 2B**). There were no studies with incomplete or biased results. The methodological quality of all selected studies remained low due to the complete lack of blinding.

### Evidence quality

**Figure 3:**
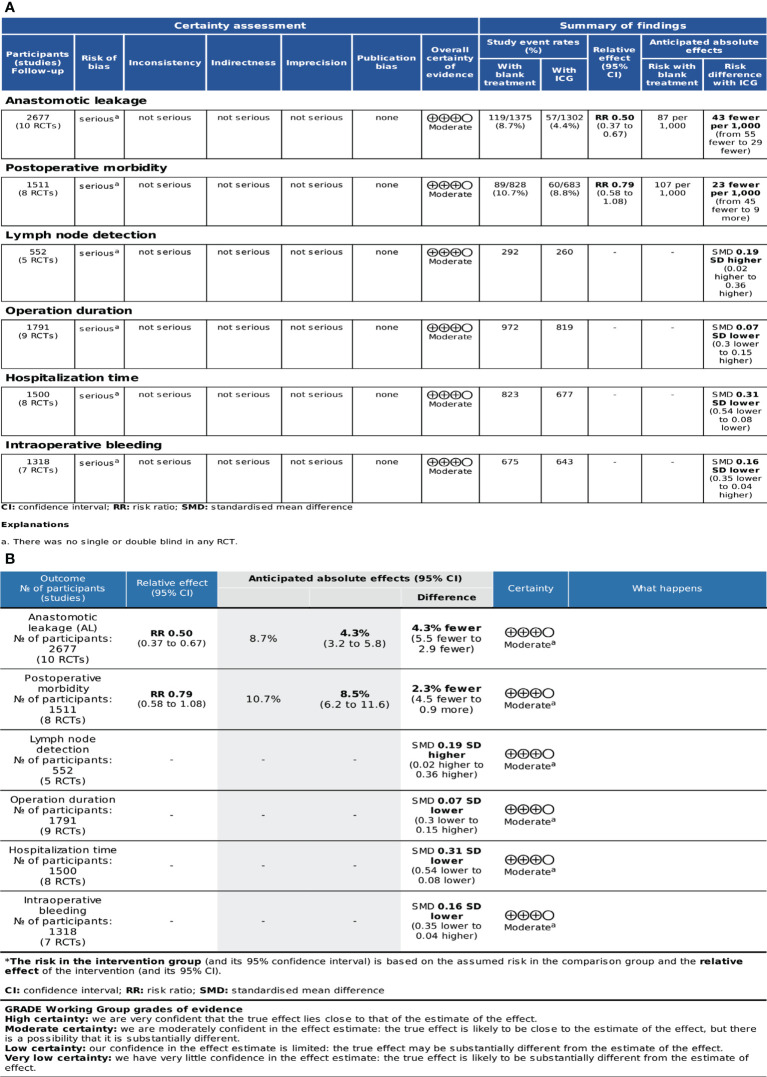
Level of quality of evidence: **(A)** GRADE evidence profile; **(B)** Summary table of findings.

The GRADEpro, which was developed by the Cochrane Collaboration Network, was used to assess the quality of evidence. A classification of the evidence was made for these studies and based on their limitations, inconsistencies, indirectness, imprecision, and publication bias. The quality of the evidence was classified as high, moderate, low, or very low. The quality of evidence was moderate for these outcome indicators due to a lack of blinding in RCTs. The profile of GRADE evidence was displayed in **Figure 3A**, and a detailed summary of the findings was shown in **Figure 3B**.

### Meta-analysis of AL

**Figure 4:**
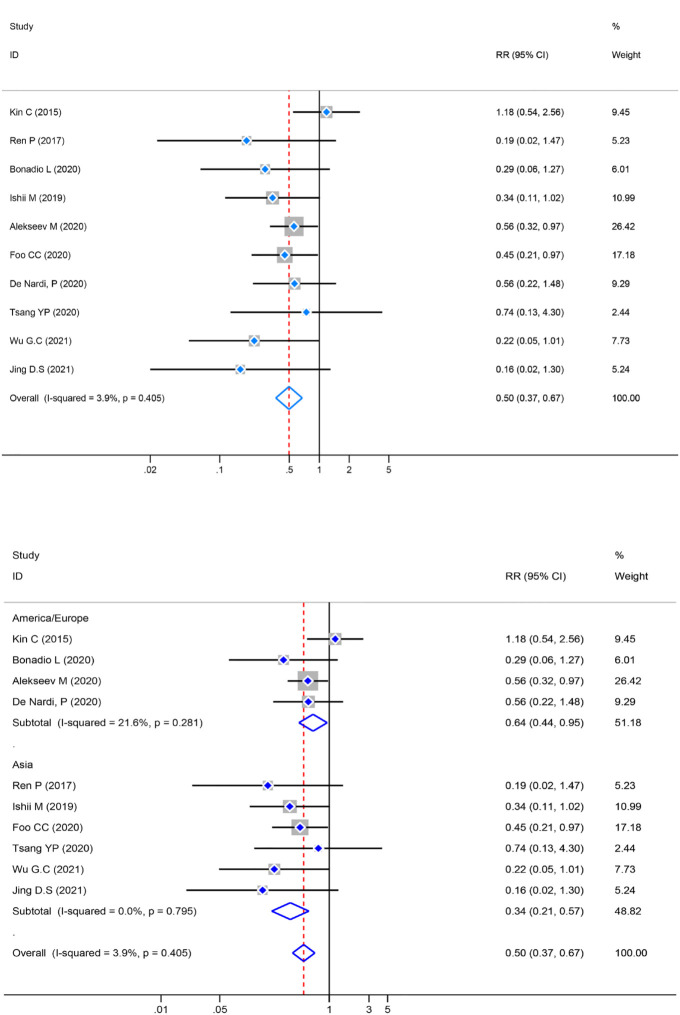
Risk ratio (RR) forest plot of AL and subgroup analysis of AL. **(A)**: Forest plot of AL. **(B)**: Subgroup analysis of AL.

Heterogeneity was examined first before the pooled analysis. Ten studies displayed low significant heterogeneity (*P*=0.405, *I* ^2^ = 3.9%) so a fixed-effect model was used to combination. The result showed a significantly lower incidence of AL [RR=0.50, 95% CI (0.37–0.67), *Z*=4.49, *P*=0.000] in the ICG group. Meanwhile, a subgroup analysis of AL was conducted by different regions. The studies were divided into two subgroups based on their geographical location: Asia and Europe/America. Four studies were reported from Europe/America [RR=0.64, 95% CI (0.44–0.95), *Z*=2.25, *P*=0.025] and six from Asia [RR=0.34, 95% CI (0.21–0.57), *Z*=4.11, *P*=0.000]. The subgroup analysis revealed statistically significant differences [RR=0.50, 95% CI (0.37–0.67), *Z*=4.49, *P*=0.000] across regions (**Figure 4**).

### Meta-analysis of postoperative morbidity

**Figure 5:**
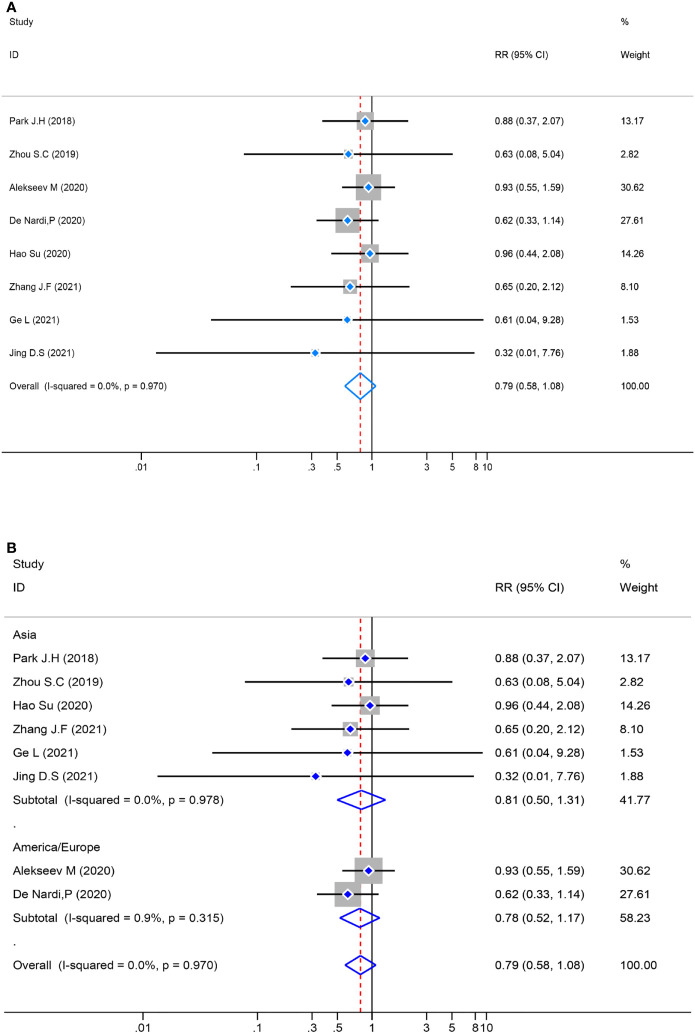
Risk ratio (RR) forest plot of postoperative morbidity and subgroup analysis of postoperative morbidity. **(A)**: Forest plot of postoperative morbidity. **(B)**: Subgroup analysis of postoperative morbidity.

The test of heterogeneity in the pooled analysis was examined first, and there was no significant heterogeneity (*P*=0.970, *I* ^2^ = 0.0%) in the eight studies. The pooled analysis of postoperative morbidity was performed by a fixed-effects model, and there was no statistically significant [RR= 0.79, 95% CI (0.58–1.08), *Z* = 1.46, *P* = 0.143] in the ICG group compared with the control group. Meanwhile, a subgroup analysis was performed, the result revealed no significant variance [RR = 0.79, 95%CI (0.58–1.08), *Z*=1.46, *P*=0.143] between the different locations (**Figure 5**).

### Meta-analysis of lymph node detection

**Figure 6:**
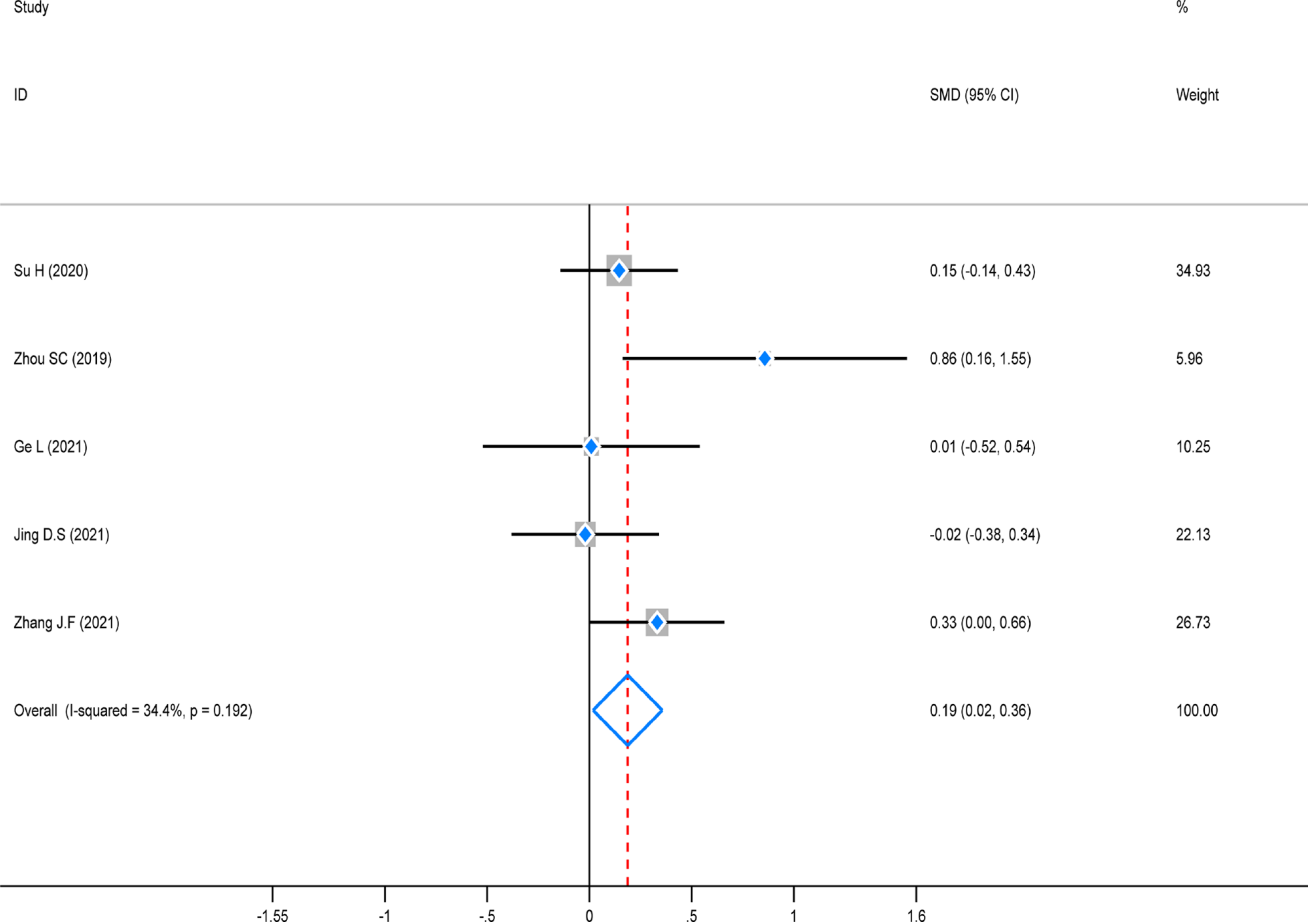
Standard mean difference (SMD) forest plot of lymph node detection.

The test of heterogeneity was first conducted before performing a pooled analysis, and there was significantly low heterogeneity across the five studies that reported lymph node detection outcomes (*P* = 0.192, *I* ^2^ = 34.4%). A fixed-effects model was used to combine the pooled analysis, and the result revealed that this indicator was noticeably higher in the ICG group than in the control group [SMD=0.19, 95% CI (0.02–0.36), *Z*=2.16, *P*=0.031]. However, a subgroup analysis was not performed due to the small number of enrolled studies (**Figure 6**).

### Meta-analysis of operation duration

**Figure 7:**
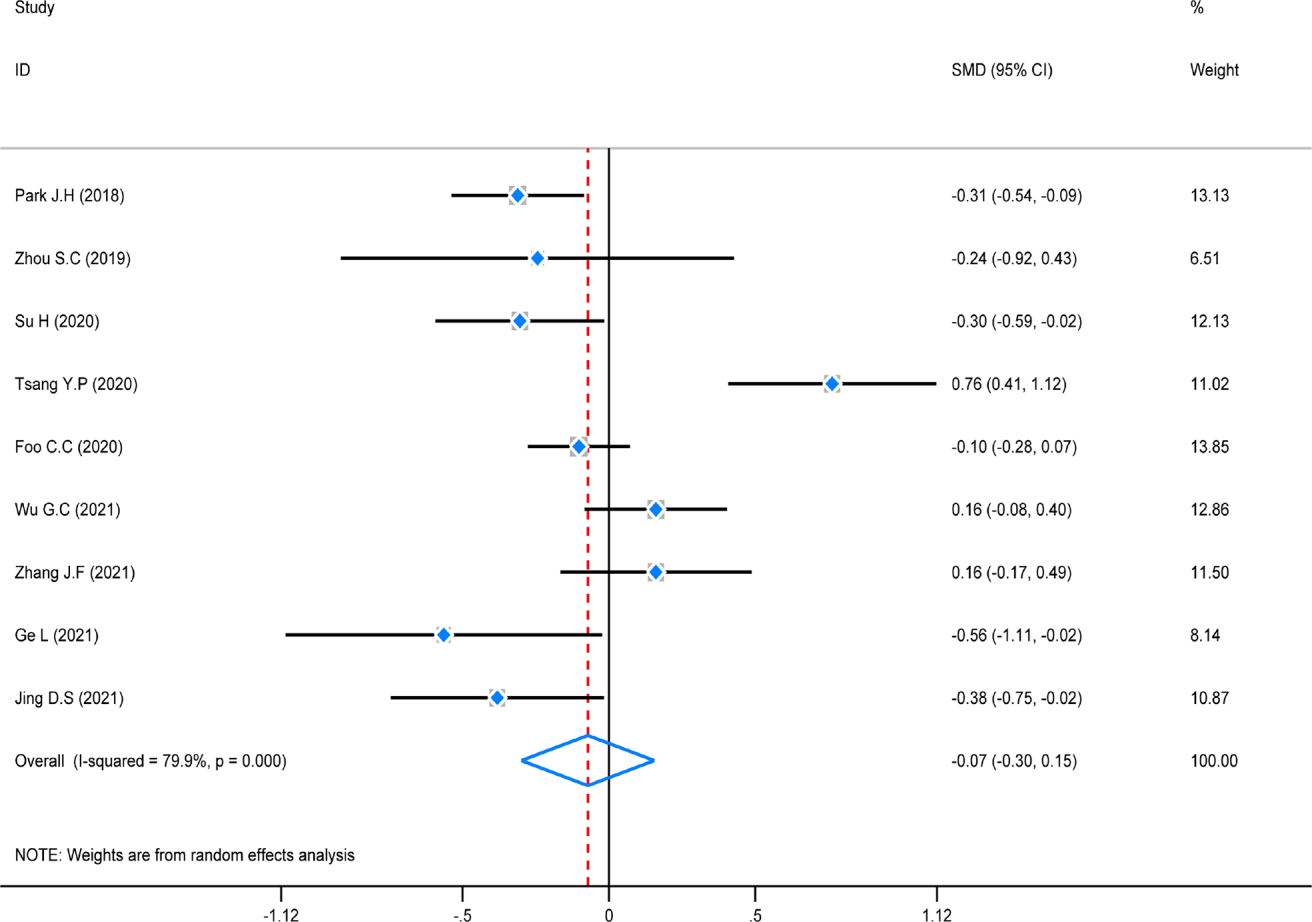
Standard mean difference (SMD) forest plot of operation duration.

The test of heterogeneity was first conducted before performing a pooled analysis, and there was obviously high heterogeneity (*P* = 0.000, *I* ^2^ = 79.9%) across the nine studies that reported operation duration. A random-effect was used to do the pooled analysis. The result revealed that there was no statistically significant difference among these studies (SMD = -0.07, 95% CI (-0.30–0.15), *Z* = 1.53, *P* = 0.126). However, a subgroup analysis was not conducted because of the same distribution (**Figure 7**).

### Meta-analysis of hospitalization time

**Figure 8:**
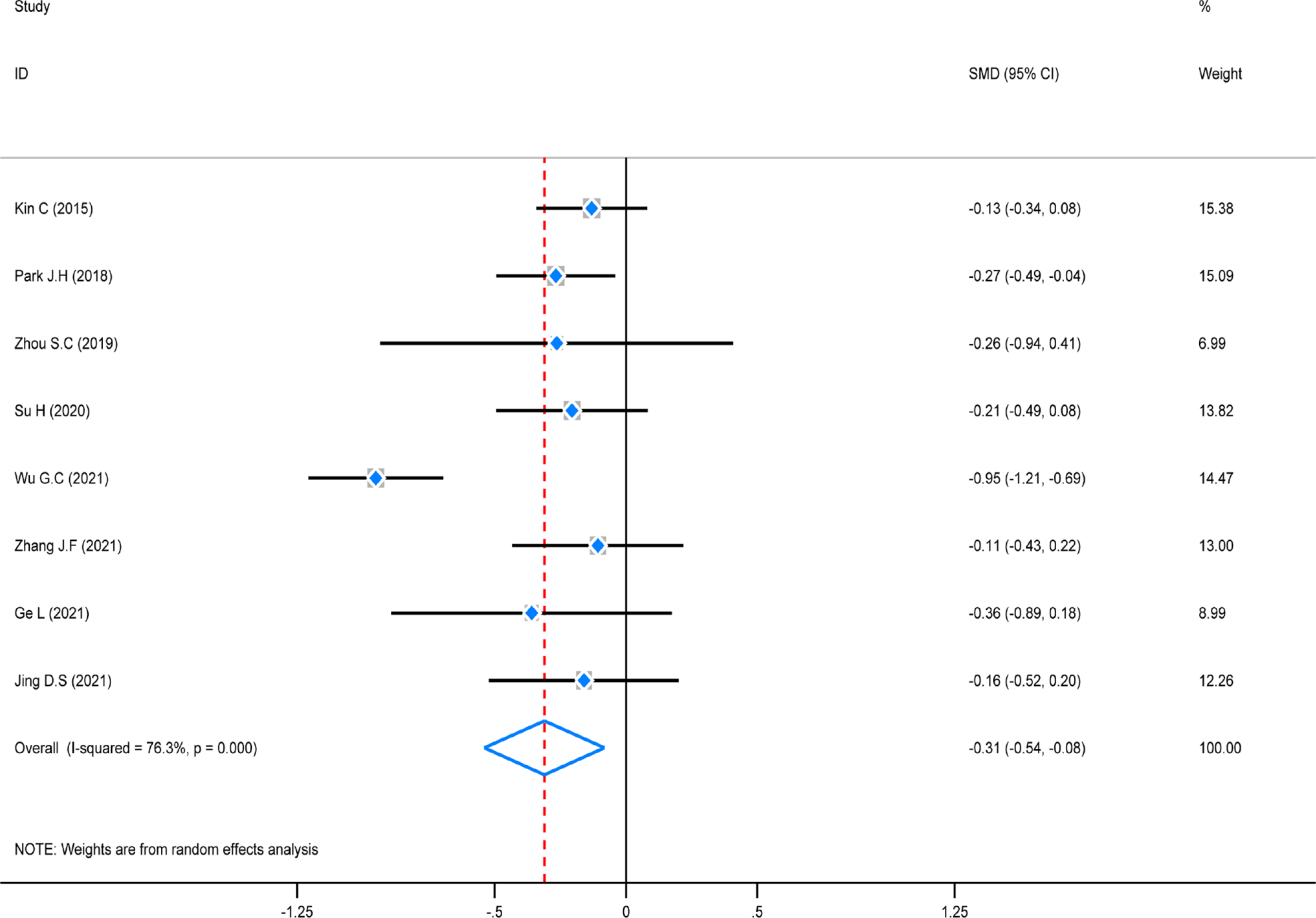
Standard mean difference (SMD) forest plot of hospitalization time.

The test of heterogeneity was first conducted before performing a pooled analysis, and the result showed that there was obviously high heterogeneity (*P* = 0.000, *I* ^2^ = 76,3%) among these studies. A random-effect model was chosen to combine, and the pooled analysis showed that hospitalization time in the ICG group was considerably decreased (SMD = -0.31, 95% CI (0.54–0.08), *Z* = 5.96, *P* = 0.000). A subgroup analysis was not carried out due to the small number of included studies coming from America/Europe (**Figure 8**).

### Meta-analysis of intraoperative bleeding

**Figure 9:**
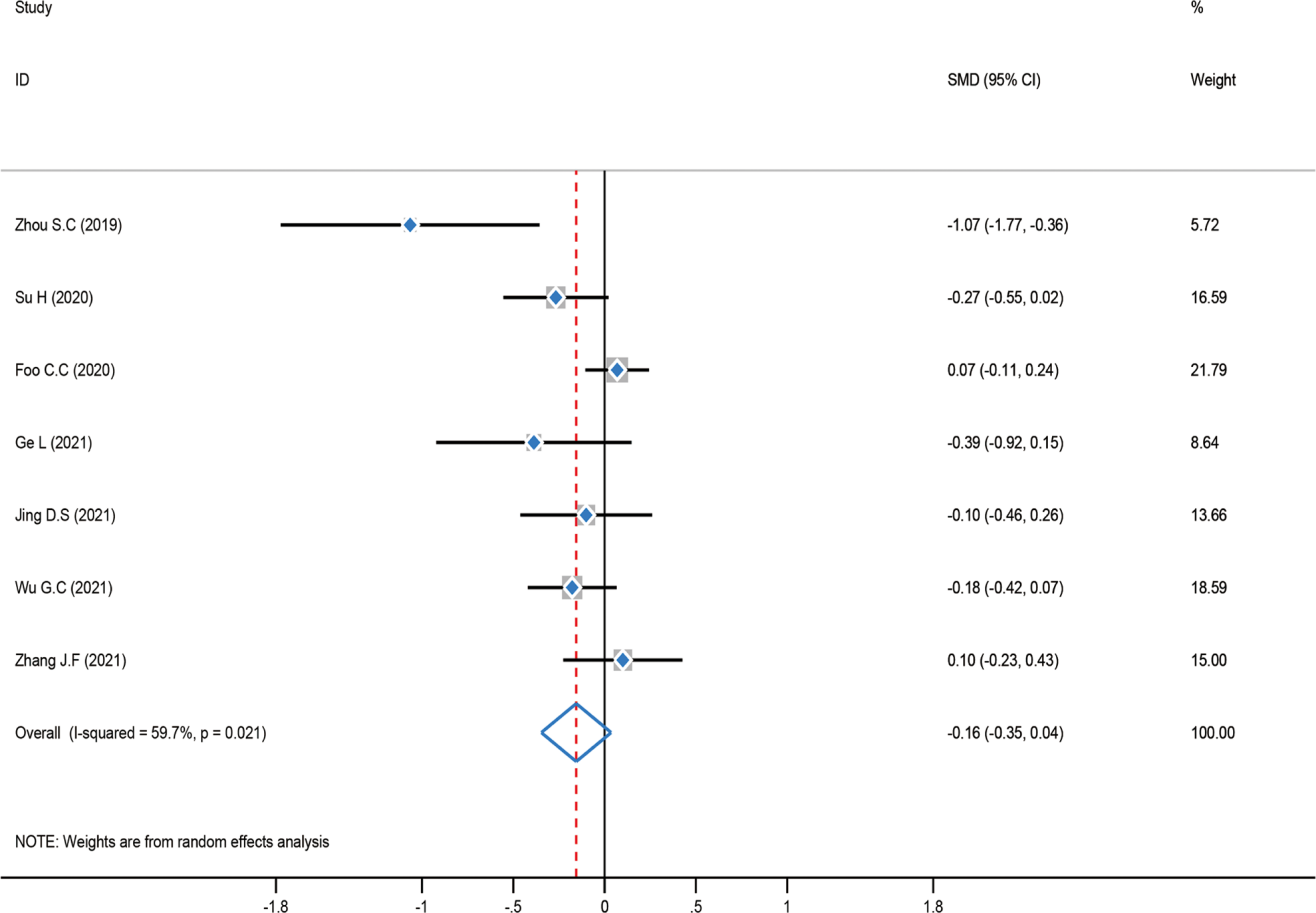
Standard mean difference (SMD) forest plot of intraoperative bleeding.

The test of heterogeneity was first examined before performing a pooled analysis, and the result showed that there was moderate heterogeneity (*P* = 0.021, *I* ^2^ = 59.7%) among these studies. Consequently, a pooled analysis was carried out using a random-effect model. The result showed that there was no statistically significant difference between the ICG group and the control group [SMD = -0.16, 95% CI (-0.35–0.04), *Z* = 1.54, *P* = 0.122]. A subgroup analysis was not carried out in this meta-analysis since intraoperative bleeding was distributed similarly across regions (**Figure 9**).

### Sensitivity analysis for robustness of pooled analysis

**Figure 10:**
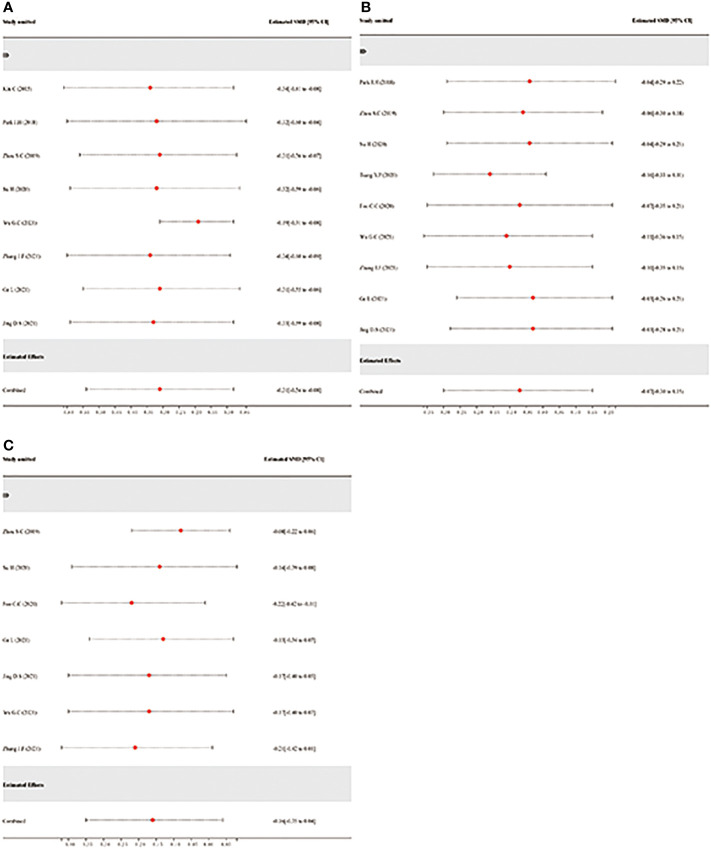
Sensitivity analysis *via* leave-one-out procedure each time. **(A)**: Sensitivity analysis of hospitalization time. **(B)**: Sensitivity analysis of operation duration. **(C)**: Sensitivity analysis of intraoperative bleeding.

The sensitivity analysis was conducted by the leave-one-out method to evaluate the robustness of the combined results (hospitalization time, operation duration, and intraoperative bleeding) in the study. The robustness of the hospitalization time may have been impacted if Wu.G.C. (21) was excluded from the study, but there were no opposing results (estimated SMD = -0.19, 95% CI: -0.31–0.08; **Figure 10A**). According to the sensitivity analysis of operation duration, Tsang Y.P. (25)’s exclusion from the study may have an impact in this study but not the opposite way around (estimated SMD = -0.16, 95% CI: -0.33–0.01; **Figure 10B**). In addition, the sensitivity analysis of intraoperative bleeding (**Figure 10C**) demonstrated that the robustness and reliability of the pooled analysis would not be significantly impacted by any study. Finally, the pooled analysis was generally reliable and robust to some degree according to all the sensitivity analysis findings.

### Contour-enhanced funnel plot to detect the potential source of publication bias

**Figure 11:**
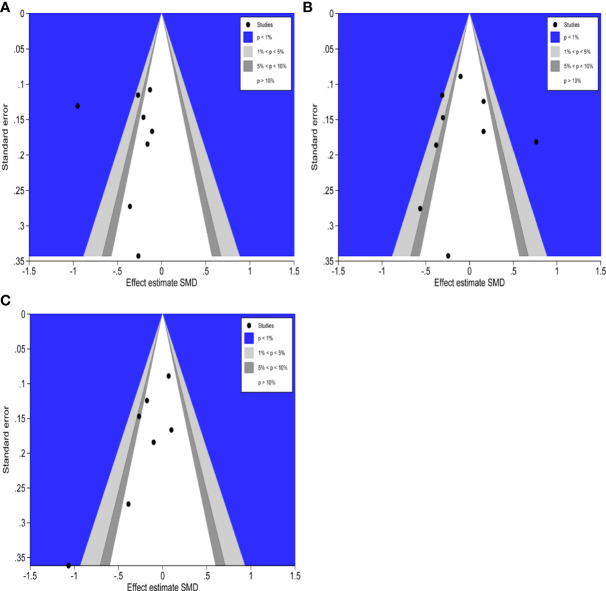
Contour-enhanced funnel plots of hospitalization time, operation duration and intraoperative bleeding. **(A)**: Contour-enhanced funnel plot of hospitalization time **(B)**: Contour-enhanced funnel plot of operation duration. **(C)**: Contour-enhanced funnel plot of intraoperative bleeding.

The counter-enhanced funnel plots were used to investigate the cause of publication bias, which contours represent statistically significant conventional milestones (*P* < 0.01, *P* < 0.05, *P* < 0.1, or *P* > 0.1). The contour-enhanced funnel plots of hospitalization time (**Figure 11A**), operative duration (**Figure 11B**), and intraoperative bleeding (**Figure 11C**), all missing RCTs were within the region of low statistical significance (P >0.1), which suggested that the asymmetry of the funnel plots was due to publication bias.

## Discussion

With the rapid progress of the modern minimally invasive techniques, the efficacy of laparoscopic colorectal surgery in the treatment of CRC has been confirmed (29–31). The surgeon would observe the abdomen clearly, peel off the lesion, and detect lymph nodes effectively under the laparoscopy. Meanwhile, due to the potential of laparoscopy to reduce postoperative pain and promote the recovery of digestive function, the impact on the abdominal cavity becomes small. Furthermore, laparoscopy makes the images larger, the surgery more precise, and decreases intraoperative bleeding and surgical injuries (7, 32, 33). However, there are still many difficulties in laparoscopic colorectal cancer, among which the main difficulty is to accurately determine the anastomosis blood flow during the operation. The primary cause of AL is the insufficient anastomotic blood flow during surgery. AL can be reduced if the operation program is revised based on anastomotic blood supplies in time (34). Currently, the perfusion of the digestive segment during laparoscopy mainly depends on the surgeon’s subjective opinion, such as monitoring the color of the anastomosis and observing the incision margin, etc. There is a possibility of AL due to errors in judgment objectively (35). Furthermore, it is still a difficulty to detect lymph nodes completely in laparoscopic colorectal surgery. It is essential to evaluate the quality of surgery by detecting lymph nodes thoroughly in operation, especially for lymph nodes, which may be ignored by using standard dissection protocols based on experience. Therefore, it is urgent to find out a technique that can accurately perform laparoscopic surgery and improve the effectiveness of surgical therapy while guiding and displaying anastomosis blood flow and lymph node detection in real time throughout the entire operation process. Therefore, it will be an important technological breakthrough to find a method that can guide and determine anastomosis blood flow and lymph node detection for laparoscopic colorectal surgery.

ICG is a mildly toxic fluorescent substance that has been used in colorectal surgery more and more in recent years to monitor intestinal perfusion and visualize the surgical area, thus the incidence of AL would effectively reduce (36, 37). From 1998 to 2003, Kudszus et al. enrolled 402 patients in a control group without ICG who had colorectal surgery and a test group of patients who underwent ICG-assisted surgery from 2003 to 2008. The test group suggested that patients with poor perfusion underwent reanastomosis with the proximal bowel free, and the final percentage of AL was observed in both the test and control groups of 7.5% (15/201) and 3.5% (7/201), which indicated that ICG reduced the incidence of AL (38). The use of ICG in colorectal anastomosis procedures is increasing, and all of them have shown good results (37, 39, 40). The application of ICG provides a new option for lymphatic tracing technology (41, 42). Lymph nodes can be detected by ICG tracing up to 65.5%–100% for CRC, which helps guide lymph node dissection and raise the probability of having more positive lymph nodes (31, 43–46). The study by Nishigori et al. found that ICG altered lymph node dissection in 23.5% (4/21) of cases, and all nodes >5 mm in diameter were identified (47).

It was found that the use of ICG in laparoscopic colorectal surgery reduced AL, enhanced lymph node detection, and reduced hospitalization time. ICG assesses anastomotic blood flow more objectively before and after intestinal anastomosis in laparoscopic colorectal surgery and reduces the incidence of AL, which is consistent with the findings of Jafari MD et al. (38). Meanwhile, ICG has some advantages in lymph node tracing, such as the ability to precisely locate the distribution of metastatic lymph nodes and anterior lymph nodes, particularly extra-regional lymph nodes, and guide intraoperative lymph node dissection, improve lymph node detection rate after surgery, and reduce the number of missed positive lymph nodes (48, 49). Furthermore, ICG can precisely locate the lesion with less intraoperative trauma during laparoscopic colorectal surgery to reduce the hospitalization time. In this study, there was no statistical difference in the postoperative morbidity, intraoperative bleeding, and operation duration between the two groups, which suggested that the application of ICG did not affect the surgical operation and postoperative recovery of the patients. In conclusion, the use of ICG in laparoscopic colorectal surgery is reliable and efficient, with the primary benefits of improving lymph node detection, reducing AL, and shortening hospitalization time.

Although strict inclusion and exclusion criteria for the meta-analysis literature were established, the following issues remain: (i) the quantity of articles was insufficient and the included literature’s quality was average; (ii) although the inclusion studies were RCTs, not all of them mentioned the use of blinding; (iii) some indicators have a significant degree of heterogeneity when combined, which could affect the article’s results; and (iv) according to the findings of the subgroup analysis, regional differences in AL outcomes may exist. These factors may have an impact on the final conclusions, which must be validated further by a large sample, multicenter, prospective randomized controlled trials.

Conclusively, ICG is dependable and effective in laparoscopic colorectal surgery, and it can be used to precisely locate the tumor and thoroughly clear the lymph node area intraoperatively, as well as to determine the blood supply to the intestine at the anastomosis, which has important practical value for reducing AL and improving lymph node detection.

## Data availability statement

The original contributions presented in the study are included in the article/supplementary material. Further inquiries can be directed to the corresponding authors.

## Author contributions

JD performed the search and drafted the manuscript. WH and YL performed the data extraction. TY and KX analyzed the data. XL and TX designed the study and amended the original draft. YL, WH, TY and KX equally involved and equally contributed into the study conduction. All authors contributed to the article and approved the submitted version.

## Funding

This study is supported by the National Natural Science Foundation of China (No. 81860854, 82260936) and the National Natural Science Foundation of China’s Post Grant Fund (No. 2018YFC170610512).

## Acknowledgment

We are grateful to the authors for providing detailed data for this meta-analysis, as well as to all colleagues in this study for their hard work.

## Conflict of interest

The authors declare that the research was conducted in the absence of any commercial or financial relationships that could be construed as a potential conflict of interest.

## Publisher’s note

All claims expressed in this article are solely those of the authors and do not necessarily represent those of their affiliated organizations, or those of the publisher, the editors and the reviewers. Any product that may be evaluated in this article, or claim that may be made by its manufacturer, is not guaranteed or endorsed by the publisher.
